# Sports facilities, socio-economic context and overweight among the childhood population in two southern European cities: a cross sectional study

**DOI:** 10.1186/s12887-019-1694-1

**Published:** 2019-09-03

**Authors:** Romana Albaladejo, Rosa Villanueva, Paloma Astasio, Paloma Ortega, Juana Santos, Enrique Regidor

**Affiliations:** 1 Departmento de Salud Pública y Materno-infantil. Facultad de Medicina, Universidad Complutense de Madrid, Ciudad Universitaria, 28040 Madrid, Spain; 2 CIBER Epidemiología y Salud Pública (CIBERESP), Madrid, Spain

**Keywords:** Sports facilities, Physical activity, Socioeconomic context, Overweight childhood

## Abstract

**Background:**

To evaluate relationship between socio-economic environment and overweight in Madrid and Barcelona, adjusting for possible confounding factors.

**Methods:**

We obtained three indicators which reflected socio-economic context, namely, unemployment rate, percentage of population with tertiary education, and percentage with a second home. The design is a cross sectional study. The association with overweight was estimated using odds ratios by multilevel logistic regression. The statistical analysis, data synthesis, or model creation was performed from the 2017. In all, 707 children from 21 districts of Madrid and 474 children from 10 districts of Barcelona were analysed.

**Results:**

In Madrid, standardised ORs for personal and family characteristics were 1.17, 1.53 and 1.57 by reference to unemployment rate and percentages of population with a university education and second home. After adjustment, only the OR obtained with unemployment rate decreased, specifically by 58%. In Barcelona, the following ORs were obtained: 1.80 with unemployment rate; 1.80 with population having a university education; and 1.86 with population having a second home. After being standardised, these ORs decreased by 14% in the case of unemployment rate, 10% in the case of population with a university education, and 9% in the case of population with a second home.

**Conclusions:**

Overweight displayed a risk gradient in Madrid and Barcelona alike. This risk of overweight is not accounted for by physical inactivity and could, in part, be due to the availability of sports facilities.

## Background

Prevalence of overweight and obesity in children and adolescents is increasing in developed countries such as Spain (31.8%) [1]. This in turn implies future health problems, such as obesity itself (70% of obese children and adolescents will be obese adults), [2] various chronic processes, [3] and, in addition, an increase in the risk of mortality due, in particular, to ischaemic heart disease, [4].

Several factors have been proposed as determinants of childhood weight and one of them is the socioeconomic context of the area of residence of individuals. The findings of a wide variety of studies in children and adolescents show that the prevalence of overweight is greater in areas with greater material deprivation or less wealth, regardless of household socio-economic position, [5–9].

Furthermore, the principal cause of weight gain is known to be an imbalance between energy intake and expenditure, [3], where regulation of food intake in sedentary individuals is different to that in active individuals, [10]. In this equilibrium there is a threshold of physical activity: under it, body weight increases even at low calorie intakes; above it, small changes in exercise will suffice to maintain weight, even when calorie consumption increases, [4].

Different epidemiological studies on children have reported a negative association between physical activity and body mass index (BMI), [5, 11, 12], as well as a direct relationship between the presence of neighbourhood sports facilities and doing physical exercise, [9, 13, 14]. The presence of neighbourhood sports facilities might thus explain the relationship between exercise and BMI: even so, the results are controversial. Whereas some authors have found an association between sports facilities or parks and a decreased risk of excess weight, [15–17], other studies have failed to observe this association, [4, 14, 18–21].

Most of the above evidence comes from research carried out in the USA, Canada, New Zealand and the United Kingdom, and yet few studies have been conducted in other European countries, where the influence exerted by socio-economic environment on weight might well differ to that seen in English-speaking countries, [2]. Accordingly, we sought to study the relationship between socio-economic environment, as reflected by three indicators, and overweight in the cities of Madrid and Barcelona, the two towns with most inhabitants in Spain, adjusting for possible confounding factors, such as age, sex, household socio-economic position, physical inactivity and availability of sports facilities.

## Methods

### Overweight and physical inactivity

Estimates of overweight and physical inactivity were drawn from the 2005 Madrid City Health Survey and the 2006 Barcelona Health Survey. In the Madrid City Health Survey, a representative sample of the population of 1057 subjects under the age of 16 was selected and the data collection was carried out between November 2004 and June 2005. In the Barcelona Health Survey, a representative sample of the population of 758 subjects under 16 years of age and the data collection was carried out between December 2005 and July 2006 [22, 23]. Individuals were selected by two-stage cluster sampling, with stratification by census districts, which constituted the first-stage units. The census districts were selected with a probability proportional to their population size, while respondents within each district were chosen by simple random selection. Interviews were conducted at the homes of the persons selected, with questionnaires being completed by one of the parents or, where this was not possible, by the person’s guardian. Children aged 6–15 years were selected for analysis purposes. In all, 707 children from 21 districts of Madrid and 474 children from 10 districts of Barcelona were analysed.

Overweight was analyzed through the body mass index (BMI, weight in kg divided by height in m^2^), based on the weight and height referred by the parents or guardians of the children. Overweight was defined according to the criteria of Cole et al. [24]. The values of BMI derived from the limits proposed for adults by the International Obesity Task Force, (this is 25 kg / m2) [IOTF, 24] are established for boys and girls between 2 and 18 years old, according to their age and sex... In the questionnaire, respondents were also asked, “Which of these possibilities best describes the frequency with which the child does some physical activity or sport in his/her free time?” In Madrid the four possible replies were: “1. No exercise”; “2. He/she does some physical or sports activity less than once a month”; “3. He/she does some physical or sports activity one or more times a month” and “4. He/she does some physical or sports activity one or more times a week”. In the case of the Barcelona survey, the answer was chosen from among 5 options: 1. Never; 2. Seldom; 3. Sometimes; 4 Often; and, 5 Every day. These responses were grouped into the following two categories: no physical activity (first and second replies in Madrid; first reply in Barcelona); and some activity (any of the other alternatives).

### Measures of household socio-economic position

Two measures of household socio-economic position were used: educational and professional qualifications of the person who contributes the most income to the household. Children were classified by the first measure as 2nd-cycle secondary or higher education and less than 2nd-cycle secondary education, and by the second measure as non-manual (managers, businessmen/women, university-qualified professionals, self-employed persons, supervisors and service sector workers) and manual workers (skilled, semi-skilled and unskilled).

### Measures of socioeconomic context of the area

To reflect the socio-economic context of Madrid and Barcelona city districts, three indicators were used: unemployment rate and the respective percentages of the population with tertiary education and a second home. The first can be considered an indicator of material deprivation and the others would be indicators of wealth.

Districts were grouped into tertiles according to the above three indicators. These data were estimated on the basis of the 2001 population census. Each respondent was then assigned to a tertile of each indicator based on his/her district of residence.

Information on the number of sports facilities in each neighbourhood was obtained from the 2005 National Census of Sports Facilities (*Censo Nacional de Instalaciones Deportivas*). Around 40% of the population practiced one or several sports in both cities. The sports facilities had an average construction age of 18.6 years in Madrid and 17.0 years in Barcelona. In Madrid, 60.8% of sports facilities were deprived 39.2% were public, while in Barcelona 59.5% were public and 40.5% private [25]. The number of sports facilities per 1000 population was estimated for each district, and each individual was assigned to a tertile according to his/her district of residence.

### Statistical analysis

The statistical analysis, data synthesis, or model creation was performed from the 2017. We evaluated the following: firstly the relationship between indicators of socio-economic environment and overweight, population characteristics and area of residence; and secondly, the relationship between population characteristics and overweight, using the chi-squared test. The association between the three indicators of socio-economic environment and overweight was assessed using the odds ratio (OR) calculated by multilevel logistic regression. Given the hierarchical structure of the data shown at two levels, i.e., individuals within districts and the possible residual correlation among persons within districts, the OR for districts’ characteristics was estimated using multilevel logit models, which included a random effect of the intercept at the origin for each neighbourhood. The models were adjusted using the SAS GLIMMIX procedure (SAS Institute 1999). We first calculated the OR adjusted for sex and age. Educational level and occupational class were then added in a second model. After adjusting for sex, age, educational level and occupational class, physical inactivity was included to determine if this substantially reduced the magnitude of the association. The percentage change in the OR after inclusion of the physical inactivity variable was then estimated according to the formula: [(physical inactivity-adjusted OR – previous OR)/ (previous OR − 1.00)] × 100. Similarly, the availability of sports facilities was added to the model adjusted for sex, age, educational level and occupational class, and the percentage change in the OR was also calculated.

## Results

The characteristics of the subjects in the two cities, according to the three variables of area of residence, are shown in Table 1. The two cities showed a similar magnitude in the socio-economic context indicators. For example, the unemployment rate was 6.0% Madrid and 6.5% in Barcelona.

**Table 1:** Sample size, study subjects’ characteristics and area of residence, and effect size according to indicators of socio-economic environment:^a^ Madrid and Barcelona city districts

Sample size (n) and study subjects’ characteristics and area of residence	Unemployment rate^b^				Percentage of population with tertiary education^b^				Percentage with a second home^b^			
	1	2	3		1	2	3		1	2	3	
*Madrid*												
n	265	232	210		254	232	221		241	247	219	
Overweight (%)	25.3	31.5	31.4	0.128	35.4	26.7	24.4	0.008	32.8	31.6	22.4	0.015
Mean age (years)	10.9	10.7	11.0	0.392	10.9	10.88	11.0	0.797	10.9	10.8	10.9	0.972
Girls (%)	44.2	51.3	42.4	0.802	43.7	47.8	46.6	0.509	44.4	46.2	47.5	0.506
Low educational level^c^ (%)	29.4	43.3	54.3	0.948	53.1	41.6	27.6	0.551	46.1	48.0	28.8	0.389
Manual occupation^c^ (%)	29.4	45.5	56.5	0.488	53.0	42.9	30.8	0.901	47.9	48.8	30.1	0.840
Physical inactivity (%)	18.9	21.6	27.6	0.025	26.8	17.7	22.2	0.200	31.5	14.2	21.5	0.007
Sports facilities/1000 pop.	2.7	1.5	1.3	0.000	1.4	2.1	2.2	0.000	1.8	1.5	2.4	0.000
*Barcelona*												
n	195	142	137		180	138	156		183	135	156	
Overweight (%)	19.2	23.9	29.2	0.034	28.3	23.2	18.2	0.029	29.0	22.2	18.2	0.019
Mean age (years)	10.4	10.3	10.7	0.586	10.6	10.4	10.28	0.590	10.6	10.41	10.28	0.597
Girls (%)	49.2	46.5	45.3	0.464	43.9	50.0	48.7	0.362	48.6	43.7	48.7	0.979
Low educational level^c^ (%)	6.1	14.0	32.3	0.000	28.7	10.4	6.3	0.000	28.0	11.2	6.3	0.000
Manual occupation^c^ (%)	21.0	42.3	54.7	0.000	52.8	37.0	19.2	0.000	52.5	37.0	19.2	0.000
Physical inactivity (%)	14.4	8.5	11.3	0.331	11.0	9.6	14.6	0.344	11.6	8.8	14.6	0.441
Sports facilities/1000 pop.	3.3	2.4	1.9	0.000	1.9	2.5	3.6	0.000	2.0	2.5	3.6	0.000

^a^Based on the unemployment rate, percentage of population with tertiary education, and percentage of population with a second home

^b^The categories according to the unemployment rate were as follows: Madrid, tertile 1 (< 11.01%), tertile 2 (11.02–13.57%) and tertile 3 (> 13.57%); Barcelona, tertile 1 (< 10.32%), tertile 2 (10.32–11.13%) and tertile 3 (> 11.13%)

The categories according to the population with tertiary education were as follows: Madrid, tertile 1 (< 15.21%), tertile 2 (15.22–30.31%) and tertile 3 (> 30.31%); Barcelona, tertile 1 (< 15.01%), tertile 2 (15.01–27.15%) and tertile 3 (> 21.15%)

The categories according to the population with a second home were as follows: Madrid, tertile1 (< 22.42%), tertile 2 (22.43–28.32%) and tertile 3 (> 28.32%); Barcelona, tertile 1 (< 19.82%), tertile 2 (19.82–26.14%) and tertile 3 (> 26.14%)

^c^Refers to the educational level and occupation of the primary household earner

* The *p*-value for subjects’ characteristics was based on the chi-square test for trend; the *p*-value for area characteristics was based on linear regression

Overweight was related, in the case of Madrid, to the percentages of population with a tertiary education (*p* = 0.008) and second home (*p* = 0.015) respectively, and in the case of Barcelona to the unemployment rate (*p* = 0.034), higher education (*p* = 0.029) and a second home (*p* = 0.019). The areas with higher proportion of the population with higher education and with a greater proportion of the population with a second home show a negative relationship with overweight. In addition, in Barcelona the areas with higher unemployment rate showed a positive relationship with overweight.

When it came to parents’ low level of education and manual occupation, there was an association in Barcelona with all three variables (*p* = 0.000).

Insofar as physical inactivity was concerned, in Madrid this was related to the unemployment rate (*p* = 0.025) and percentage of a second home (*p* = 0.007), whereas in Barcelona there was no relationship with any of the 3 environmental variables.

Lastly, the number of sports facilities per 1000 inhabitants was associated, both in Madrid and Barcelona, with the three area variables (*p* = 0.000).

Table 2 shows the prevalence of overweight in relation to the personal variables, variables of household socio-economic position and variables of physical inactivity, for the two cities. In Madrid and Barcelona alike, the prevalence of overweight was highest among the youngest children (*p* = 0.000 and *p* = 0.000 respectively), and in Madrid, in addition, among males (*p* = 0.013).

**Table 2:** Percentage frequency of overweight by age, sex, household socio-economic characteristics and obesity risk behaviours: Spain

Characteristics	Madrid		Barcelona	
	Sample size (n)	Overweight	Sample size (n)	Overweight
Age (years)				
6–10	314	37.3	228	31.7
11–15	393	22.6	246	15.9
*p*-value		0.000		0.000
Sex				
Boy	382	32.7	250	25.3
Girl	325	24.9	224	21.5
*p*-value		0.023		0.335
Educational level^a^				
High	412	26.0	393	23.2
Low	295	33.6	81	29.0
*p*-value		0.942		0.304
Occupation^a^				
Non-manual	402	26.4	297	22.3
Manual	305	33.2	177	25.6
*p*-value		0.121		0.418
Physical inactivity				
No	549	28.8	411	24.7
Yes	158	30.4	63	19.6
*p*-value		0.697		0.422

^a^Refers to the educational level and occupation of the primary household earner

Table 3 lists the ORs with their confidence intervals for overweight according to the three area indicators, for both cities. In Madrid, after adjustment for sex, age, educational level and occupational class, the ORs for the districts situated in the tertile with the worst socio-economic context were: 1.17 (0.74–1.86) by reference to the unemployment rate; 1.53 (1.00–2.32) by reference to the percentage population with a university education; and 1.57 (1.02–2.42) by reference to the percentage of the population with a second home. Adjustment for physical inactivity did not greatly reduce the magnitude of the OR. Adjustment for availability of sport facilities similarly failed to reduce the magnitude of the OR, except when the indicator of socio-economic context was the unemployment rate, in which case the magnitude decreased by 58% (from 1.17 to 1.07). In Barcelona, after adjusting for sex, age, educational level and occupational category, the ORs for the districts situated in the tertile having the worst socio-economic context were: 1.80 (1.01–3.22) by reference to the unemployment rate; 1.80 (1.00–3.23) by reference to the percentage of the population with a university education; and 1.86 (1.04–3.33) by reference to the percentage of the population with a second home. Adjustment for physical inactivity did not greatly reduce the magnitude of the OR. Adjustment for availability of sport facilities reduced the magnitude of the OR in the tertile having the worst socio-economic context: by 14% when the indicator was the unemployment rate (from 1.80 to 1.69); by 10% when the indicator was the percentage of the population with a university education (from 1.80 to 1.72); and by 9% when the indicator was the percentage of the population with a second home (from 1.80 to 1.79).

**Table 3:** Odds ratio (95% confidence interval) for overweight, by indicators of socio-economic environment: Madrid and Barcelona

Indicators of socio-economic environment	Adjusted for age and sex		Adjusted for age, sex, and socio-economic position^a^		Adjusted for age, sex, socio-economic position and physical inactivity		Adjusted for age, sex, socio-economic position and sports facilities	
	OR	95% CI	OR	95% CI	OR	95% CI	OR	95% CI
Madrid								
Unemployment rate								
Less than 11.01%	1.00		1.00		1.00		1.00	
11.02 to 13.57%	1.34	0.87–2.05	1.25	0.81–1.95	1.26	0.81–1.96	1.16	0.68–1.99
Higher than 13.57%	1.31	0.84–2.02	1.17	0.74–1.86	1.16	0.73–1.85	1.07	0.61–1.91
Population with a university education								
Higher than 30.31%	1.00		1.00		1.00		1.00	
15.22 to 30.31%	1.11	0.72–1.71	1.04	0.67–1.61	1.05	0.68–1.63	1.04	0.67–1.61
Less than 15.21%	1.65	1.10–2.48	1.53	1.00–2.32	1.52	1.00–2.32	1.50	0.96–2.35
Population with a second home								
Higher than 28.32%	1.00		1.00		1.00		1.00	
22.43 to 28.32%	1.54	1.01–2.36	1.45	0.94–2.33	1.47	0.95–2.27	1.43	0.89–2.28
Less than 22.42%	1.65	1.08–2.52	1.57	1.02–2.42	1.56	1.01–2.40	1.54	0.79–3.00
Barcelona								
Unemployment rate								
Less than 10.31%	1.00		1.00		1.00		1.00	
10.32 to 11.13%	1.30	0.74–2.27	1.23	0.69–2.17	1.20	0.68–2.14	1.17	0.65–2.13
Higher than 11.13%	2.00	1.16–3.44	1.80	1.01–3.22	1.78	0.99–3.18	1.69	0.90–3.18
Population with a university education								
Higher than 27.15%	1.00		1.00		1.00		1.00	
15.03 to 27.15%	1.41	0.77–2.58	1.35	0.73–2.50	1.34	0.72–2.47	1.32	0.69–2.52
Less than 15.03%	1.99	1.15–3.46	1.80	1.00–3.23	1.77	0.99–3.19	1.72	0.88–3.37
Population with a second home								
Higher than 26.14%	1.00		1.00		1.00		1.00	
19.83 to 26.14%	1.34	0.73–2.47	1.28	0.69–2.38	1.26	0.68–2.35	1.25	0.65–2.41
Less than 19.82%	2.05	1.18–3.56	1.86	1.04–3.33	1.84	1.03–3.29	1.79	0.93–3.48

^a^The variables of socio-economic position were the educational level and social class of the primary household earner

## Discussion

This study sought to assess the influence exerted on overweight by socio-economic context, measured by reference to the unemployment rate, and the percentages of the population having a tertiary education and second home respectively, in view of the fact that earlier studies have shown that, regardless of household characteristics, children and adolescents living in deprived neighbourhoods tend to have a higher body weight, [5, 26].

As a first step, a descriptive study was conducted to measure the relationship between individual and socio-economic factors. Since a strong association was found between the latter indicators and the number of neighbourhood sports facilities (Table 1, *p* < 0.000), it was decided to include these in the adjustments in the regression analysis. While previous studies undertaken in the USA, Canada and the United Kingdom [14, 27] have shown that people living in underprivileged areas are more inactive, it is equally likely that they have fewer sports facilities and green areas, [14, 28], and moreover, that this availability varies from city to city, [14].

Overweight in Madrid displayed a risk gradient, except vis-à-vis the unemployment rate, which was highest in the intermediate tertile (Table 3). In Barcelona, this increased risk was again in evidence, especially in the case of the population that had a second home (Table 3). Physical inactivity would not account for this risk of overweight in either of the two cities: in Madrid, the availability of sports facilities reduced this risk by 58% for the upper tertile of the unemployment rate; and in Barcelona, the availability of sports facilities accounted for around 10% of overweight with respect to all three indicators.

A previous study, undertaken in Canada, [5], reported a relationship between a socio-economic environmental indicator, i.e., the unemployment rate, and weight, an association also reported for other indicators by a number of studies, [5, 29–31]. Although it is reasonable to assume that one of the mechanisms whereby socio-economic level influences overweight would be greater physical inactivity, [13, 14, 27], a certain degree of controversy nevertheless surrounds this contention, [5]. In this regard, while previous studies have found that the number of neighbourhood sports facilities is related to lower weight, [15, 16, 32] and that the most underprivileged neighbourhoods have fewer centres at which people can do physical exercise, [33], other studies have failed to observe this relationship, [34]. It should be borne in mind here that a range of factors, including the availability of foot or cycle-paths leading to such sports facilities, [35], the distance to be covered, [17], their ease of access, [21, 36], the quality of the sports centres themselves, and the safety of the neighbourhoods in which they are situated, [3, 37–39], could exert an influence, giving rise to these differences in results.

It should be stressed that, in general, socio-economic factors were better indicators of risk in the case of Barcelona than in that of Madrid. This might be due to the administrative reorganisation of the city of Barcelona, implemented in 2007, which divided it into neighbourhoods created on the basis of sociological criteria, [40]. In Madrid, in contrast, districts are administrative divisions of the city; hence, rather than being constructs devised for the purpose of grouping together neighbouring areas with similar characteristics, they are units having a certain degree of internal homogeneity, [40].

Furthermore, it should be borne in mind that that socio-economic environmental determinants have less influence than do individual determinants on risk of overweight [5], for the simple reason that, because they are drawn from census data, they probably yield an underestimate of the real effects, due to a smaller degree of variability in the population and less opportunity for differential misclassification bias.

Insofar as the strong points of this study are concerned, mention should be made, firstly, of the fact that the data were drawn from 2 random samples of the two largest cities in Spain, and that use was made of routine census data collected by government authorities for public health purposes. Moreover, the study assessed both the individual factors (age, sex, socio-economic position and physical inactivity), and the environmental factors of wealth and poverty (unemployment rate, population with tertiary education, and population having a second home), and integrated the relationship between the two types of factors and the weight of children and adolescents, as well as the presence of neighbourhood sports facilities, by means of multilevel analysis.

A potential limitation of our study is its cross-sectional design, which does not take the temporal relationship among the variables into account, though it is highly unlikely that overweight among children would be determined by the neighbourhood in which they resided. It should likewise be noted that the BMI data were reported by the interviewees, who might not have measured the weight accurately, [41]. Various studies on obesity in children have used weight and height referred by the person responsible for the child [42, 43] and in other countries [44, 45]. In a study conducted in Spain, the BMI with information declared by the parents showed a sensitivity of 78% and a specificity of 96% [46] and in a study conducted in Germany the authors obtained sensitivities between 78 and 85% and specificities close to 100%. In any case, we cannot rule out an information bias in the measurement of overweight in our study, although there is no reasons to think that misclassification of subjects be differential with respect to the socioeconomic context indicators used.

In our study, the questionnaire was validated in 50 interviewees in the Madrid City Health Survey and in 18% of those interviewed in the Barcelona Health Survey, and it was found that information on weight and height had high reliability [22, 23] Similarly, the possibility of there being unknown errors of measurement in the collection of data on physical inactivity should not be ruled out; even so, there is no need to suppose that such errors would differ in terms of neighbourhood of residence. The use of a comprehensive population source, such as the census, for the socioeconomic characterization of the population, has the advantage that there is less probability of an information bias in this exposure measure. On the other hand, being able to reflect the great socioeconomic heterogeneity may have led to an underestimation of the effect.

Finally, as in other studies, future research is called for to study the working mechanisms through which neighbourhood social environment exerts its effect on BMI, [47].

## Conclusions

We study the relationship socio-economic environment, and overweight in two southern European cities: overweight displayed a risk gradient in Madrid and Barcelona. That is, the areas of these cities with the best socioeconomic indicators show the lowest prevalence of overweight and the areas with the worst socioeconomic indicators showed the highest prevalence of overweight among the childhood population. Although in our study we have not shown that physical inactivity is a determining factor in overweight, there is a decrease in the risk of overweight in relation to the availability of sports facilities in the two cities.

## Data Availability

The datasets used and/or analysed during the current study are available from the corresponding author on reasonable request.
